# Activated Effects of Parathyroid Hormone-Related Protein on Human Hepatic Stellate Cells

**DOI:** 10.1371/journal.pone.0076517

**Published:** 2013-10-07

**Authors:** Fen-Fen Liang, Cui-Ping Liu, Li-Xuan Li, Min-Min Xue, Fang Xie, Yu Guo, Lan Bai

**Affiliations:** 1 Guangdong Provincial Key Laboratory of Gastroenterology, Department of Gastroenterology, Nanfang Hospital, Southern Medical University, Guangzhou, Guangdong Province, China; 2 Department of Huiqiao Building, Nanfang Hospital, Southern Medical University, Guangzhou, Guangdong Province, China; Haassah Medical Center, Israel

## Abstract

**Background & Aims:**

After years of experiments and clinical studies, parathyroid hormone-related protein(PTHrP) has been shown to be a bone formation promoter that elicits rapid effects with limited adverse reaction. Recently, PTHrP was reported to promote fibrosis in rat kidney in conjunction with transforming growth factor-beta1 (TGF-β1), which is also a fibrosis promoter in liver. However, the effect of PTHrP in liver has not been determined. In this study, the promoting actions of PTHrP were first investigated in human normal hepatic stellate cells (HSC) and LX-2 cell lines.

**Methods:**

TGF-β1, alpha-smooth muscle actin (α-SMA), matrix metalloproteinase 2 (MMP-2), and collagen I mRNA were quantified by real-time polymerase chain reaction (PCR) after HSCs or LX-2 cells were treated with PTHrP(1–36) or TGF-β1. Protein levels were also assessed by western-blot analysis. Alpha-SMA were also detected by immunofluorescence, and TGF-β1 secretion was measured with enzyme-linked immunosorbent assay (ELISA) of HSC cell culture media.

**Results:**

In cultured human HSCs, mRNA and protein levels of α-SMA, collagen I, MMP-2, and TGF-β1 were increased by PTHrP treatment. A similar increasing pattern was also observed in LX-2 cells. Moreover, PTHrP significantly increased TGF-β1 secretion in cultured media from HSCs.

**Conclusions:**

PTHrP activated HSCs and promoted the fibrosis process in LX-2 cells. These procedures were probably mediated via TGF-β1, highlighting the potential effects of PTHrP in the liver.

## Introduction

Parathyroid hormone-related protein (PTHrP) was first identified from cancers that caused hypercalcemia, but over 25 years of study, it has been demonstrated to work as a multifunctional cytokine [1]. However, studies of PTHrP have mainly focused on bones and tumors. Experiments have demonstrated that PTHrP promotes bone formation and is an excellent osteogenic promoter that exerts rapid effects and few adverse reactions, even after years of clinical treatments for osteoporosis (OP) [2], [3], [4], [5]. Recently, PTHrP was reported to promote renal fibrogenesis, with the cooperation of TGF-β1 (transforming growth factor-β1), EGF (endothelial growth factor), and VEGF (vascular endothelial growth factor) [6]. TGF-β1 is a powerful fibrosis promoter and plays a central role in many fibrosis processes, including liver fibrosis.

During chronic liver disease, hepatic stellate cells (HSCs) is a principal fibrogenic cell type that contributes to collagen accumulation [7]. Activation of HSCs is a key event in hepatic fibrosis, where they acquire contractility and the extracellular matrix (ECM) changes as they transform to myofibroblast-like cells [8]. These cells express the myofibroblast marker α-smooth muscle actin (α-SMA) [9], [10], and synthesize fibrillar collagens. The initiation and persistence of HSC activation is regulated by many signaling molecules, including TGF-β1 [11]. HSC activation can strongly produce TGF-β1 to maintain its elevated level, and TGF-β1 then activates and recruits more myofibroblasts to the injured liver [12]. This resulting in enhanced deposition of collagens into the interstitial spaces, which finally impairs liver function [13].

PTHrP it is normally produced in every body tissue and organ, including the liver [14], [15]. Previous studies demonstrated that PTHrP was markedly induced in hepatocytes during endotoxemia and caused hepatic acute phase response [16], [17]. These results suggest that PTHrP may be an additional cytokine involved in liver disease, but the exact effects of PTHrP on liver tissue is poorly understood. Some chronic liver disease patients experience endotoxemia. The hypothesis of the importance of endotoxins in liver damage was first published in 1975 [18], and the critical role of endotoxin in acute and chronic liver disease is now well accepted and correlated with the disease severity [19]. However, the exactly effects of PTHrP in normal liver or in endotoxemia have not yet been evaluated.

Datas regarding the effects of PTHrP on the liver or hepatic diseases are rare. The aim of the present study was to obtain a basic understanding of the effects of PTHrP in normal HSCs and the activated LX-2 cell line. We show here for the first time that PTHrP activates HSCs and promotes the fibrosis process of LX-2 cells, suggesting a role of the TGF-β1 system in promoting detrimental effects.

## Materials and Methods

### Cell culture

HSC and LX-2 cell lines were obtained from the cell bank of Sun Yat-sen University, Guangzhou, China. All these cells were growth in Dulbecco's modified eagle medium (DMEM, from Gibco,USA) with 10% FCS(fetal calf serum, from Gibco) in 5% CO_2_ at 37°C. For all of the experiments, subconfluent cells (80%) were incubated in 6-well dishes with either various PTHrP (1–36) (Bachem, Bubendorf, Switzerland, H-3208) concentrations (0.1, 1, 10, 100 nM), or TGF-β1 (ProSpec, USA, CYT-716) of 1 ng/ml as positive control, or 100 nM PTHrP with 1 ng/ml TGF-β1 in the presence in DMEM with 2% FCS for different time periods (6, 12, 24, 48 h).

### Western blot analysis

Cells were harvested in 0.2 ml of RIPA lysis buffer (Beyotime Biotech, Nantong, China) with protease inhibitors (Roche, Switzerland) and centrifuged with 12000 rpm for 20 min. The supernatants were assayed for protein concentration (Beyotime Biotech, Nantong, China). Protein samples were heated at 100°C for 5 min before loading and 30 µg of the samples were subjected to 10% sodium dodecyl sulphate-polyacrylamide gel electrophoresis (SDS-PAGE) and then transferred to poly-vinylidene fluoride (PVDF) membranes. Next, membranes were blocked with 5% skimmed milk powder in TBST buffer (20 mM Tris, 500 mM NaCl, and 0.1% Tween-20) for 1 h at room temperature with gentle shaking. Membranes were then incubated overnight at 4°C with various primary antibodies. The following primary antibodies were used: 1∶1000 mouse polyclonal anti-TGF-β1 (Abcam, Cambridge, UK, ab64715), 1∶1000 rabbit polyclonal anti MMP-2 (Sigma-Aldrich, St. Louis, MO, SAB4501891), 1∶1000 rabbit polyclonal anti-collagen I (Abcam, ab34710), 1∶1000 rabbit polyclonal anti-α-SMA (Santa Cruz Biotechnology, Santa Cruz, CA, sc-130619).The membranes were washed with TBST buffer and incubated in the appropriate peroxidase-conjugated secondary antibody solution at a 1∶5000 dilution (Zhongshan Biotech, Beijing, China) before they were finally developed with enhanced chemiluminescence (Millipore Corporation, USA, WBKLS0100). The density of the individual bands was then quantified using a densitometric scanner with Gel-pro Analyzer (Media Cybernetics, USA).

### mRNA expression analysis

Total RNA was isolated from HSCs and LX-2 cells with TRIzol (TaKaRa Bio, Japan). cDNA was synthesized using the Revert Aid First Stand cDNA Synthesis Kit(Fermentas, EU, #K1622) using 2 µg total RNA primed with random hexamer primers, following the manufacturer's instructions. The single-stranded cDNA was amplified by comparative quantitative real-time RT-polymerase chain reaction (PCR) using SYBR green Master Mix kit (Roche, USA, Cat. No. 04887352001) on an Roche LightCycler 480. Primers were as follows: TGF-β1, (Forward) 5′- ACC TGA ACC CGT GTT GCT CT -3′ and (Reverse) 5′- CTA AGG CGA AAG CCC TCA AT -3′; MMP-2, (Forward) 5′-GTA TTT GAT GGC ATC GCT CA -3′ and (Reverse) 5′- CAT TCC CTG CAA AGA ACA CA -3′; collagen I, (Forward) 5′-GAA CGC GTG TCA TCC CTT GT -3′ and (Reverse) 5′- GAA CGA GGT AGT CTT TCA GCA ACA -3′; α-SMA, (Forward) 5′-TCG CAT CAA GGC CCA AGA AA -3′ and (Reverse) 5′-GCT TCA CAG GAT TCC CGT CTTA -3′; GAPDH, (Forward) 5′- TGC ACC ACC AAC TGC TTA GC -3′ and (Reverse) 5′- GGC ATG GAC TGT GGT CAT GAG -3′. The cycles for PCR were as follows: one cycle of 95°C for 10 minutes, 45 cycles of 15 seconds at 95°C, 1 minute at 62°C and a final 1 minute at 72°C. The mRNA expression levels of the target genes were normalized to GAPDH.

### Immunofluorescence

Cells were plated on glass coverslips in 12-well culture dishes and grown to approximately 50% confluence to promote cell adherence of cells. After stimulation with PTHrP, the cells were then washed twice with cold phosphate-buffered saline (PBS) and then fixed in 4% paraformaldehyde in PBS for 10 minutes. After fixation, cells were washed twice with PBS and then permeabilized with PBS containing 0.1% Triton X-100 for 15 minutes. They were then washed three times with PBS and incubated with blocking solution (5% BSA in PBS) for 30 minutes. Primary antibody α-SMA (Santa Cruz Biotechnology, sc-130619, diluted 1∶200 in blocking solution) was incubated with the cells over night at 4°C. After three washes with PBS, cells were incubated with appropriate fluorescein-labeled IgG (Vector Laboratories, diluted 1∶500 in blocking buffer) for 1 h. Cells were washed three times with PBS and then stained with 5 µg/ml DAPI (4′,6-diamidino-2-phenylindole, Beyotime Biotech, Nantong, China) for 2 minutes and washed three times with PBS. Cells were viewed with a Nikon Eclipse E600 fluorescence microscope.

### Cell-conditioned medium protein assay

TGF-β1 protein was measured in the HSC-conditioned medium after treatment with PTHrP(1–36) (10–100 nM) for 24–48 h, using a commercial enzyme-linked immunosorbent assay (ELISA, eBioscience, San Diego, CA, E13702-107) following the manufacturer's instructions. Total TGF-β1 was determined in 100 µl of the cell-conditioned medium (stored at −80°C). Inactive TGF-β1 was converted to the active form by incubating these cell culture supernatants with 1 N HCl for 10 min, followed by neutralization with 1 N NaOH. Protein content was determined by the bicinchoninic acid (BCA) method (Pierce). TGF-β1 concentrations were quantified by comparison with a standard curve of human TGF-β1.

### Statistical analysis

Data are presented as mean ± standard error of the mean (S.E.M.) based on experiments repeated in triplicate. Multiple comparisons were analyzed using one-way analysis of variance (ANOVA) with Statistical Package for the Social Sciences (SPSS) 13.0 software (Chicago, IL). Probability (p)-values less than 0.05 were considered statistically significant.

## Results

### Increased production of α-SMA in HSCs and LX-2 cells after treatment with PTHrP(1–36)

**Figure 1:**
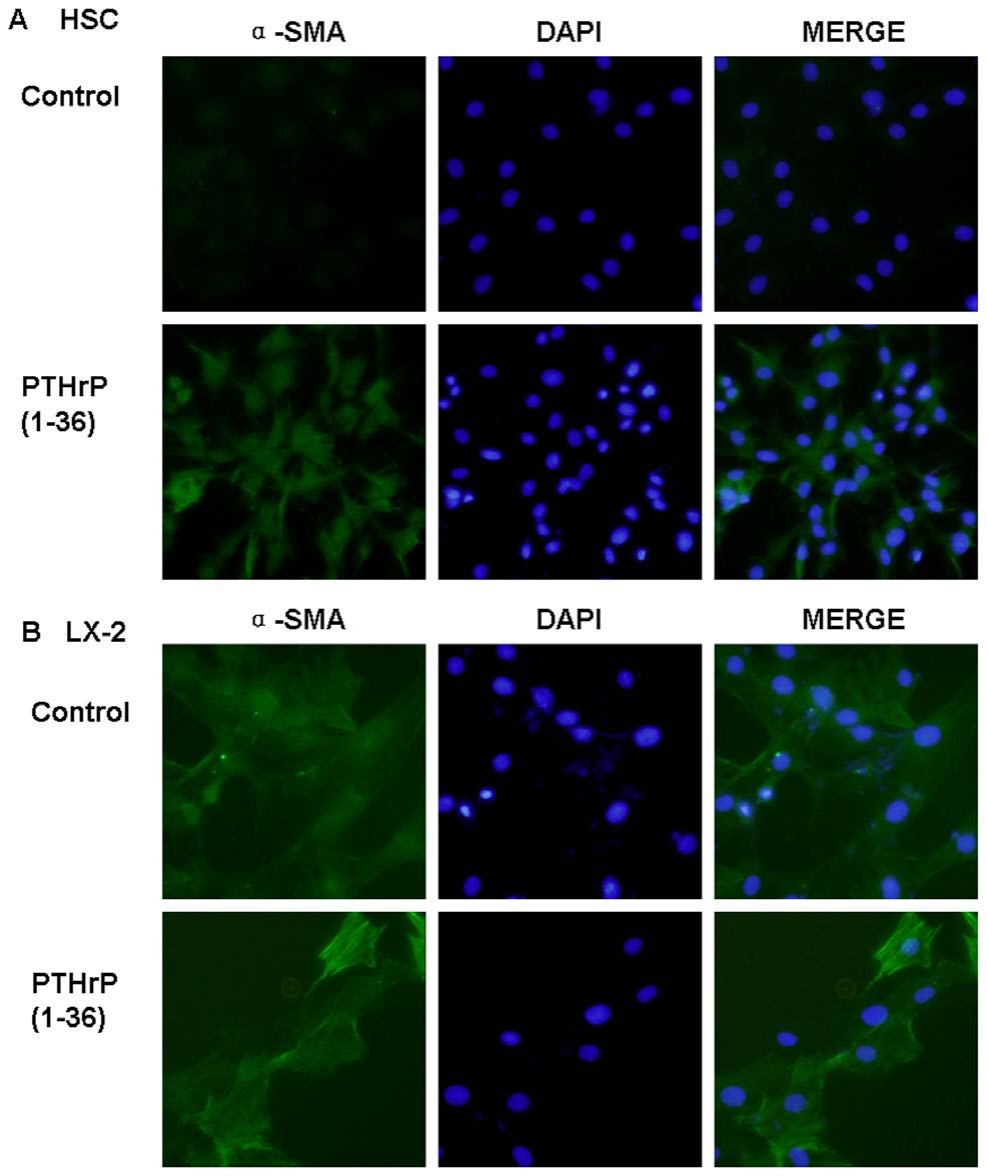
Alpha -SMA immunofluorescence. Alpha –SMA was detected in HSCs treated with 100 nM PTHrP(1–36) for 48 h (A), and stronger expression was found in LX-2 cells (B). Cells were counterstained with DAPI to identify nuclei.

**Figure 2:**
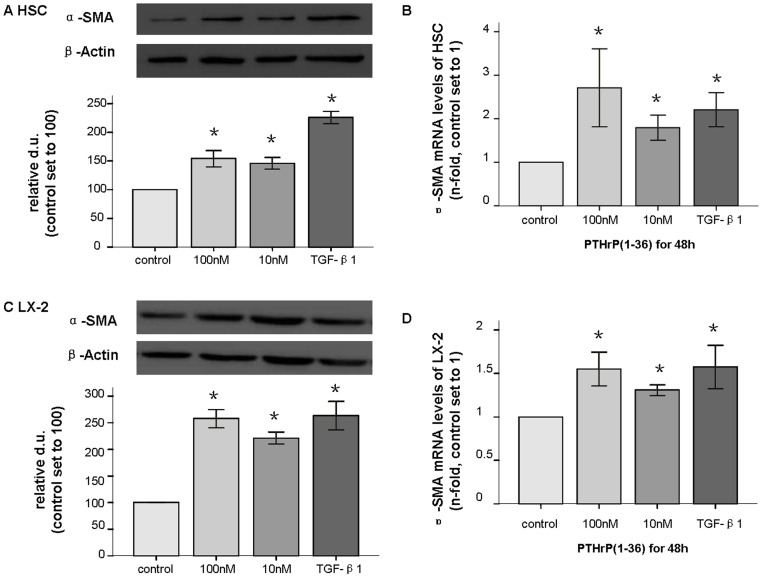
Increased production of α-SMA in HSC and LX-2 cells after PTHrP(1–36) treatment. After treatment with 100(1–36) for 48 h, the α-SMA protein (43 kd) levels assessed by western blot (A) and mRNA levels measured by q-PCR were increased in HSCs (B). The same patterns were observed in LX-2 (C and D). * p<0.05 compared to the control group.

To explore the possible effects of PTHrP in HSCs, we performed immunostaining for α-SMA. After incubation with 100 nM PTHrP(1–36). HSCs stained strongly for α-SMA, and LX-2 staining significantly increased compared with control (Figure 1). After treated with various concentrations (0.1–100 nM) PTHrP(1–36) for different time periods (6–48 h), levels of α-SMA mRNA (by q-PCR) and protein (western-blot) were found to be increased at 10–100 nM for 24–48 h in both types of cells, with 1.3- to 3- fold increases (Figure 2). At 10–100 nM PTHrP for 6–12 h, both cell types showed 0.9- to 1.1-fold increases, but there were no significant difference in these groups. Similar results were obtained for the 0.1–1 nM PTHrP groups. The strong expression of α-SMA, which is a myofibroblast marker, showed that PTHrP activates HSCs. All experiments were repeated at least three times.

### PTHrP(1–36) induced MMP-2 and collagen I mRNA and protein production in HSCs and LX-2 cells

**Figure 3:**
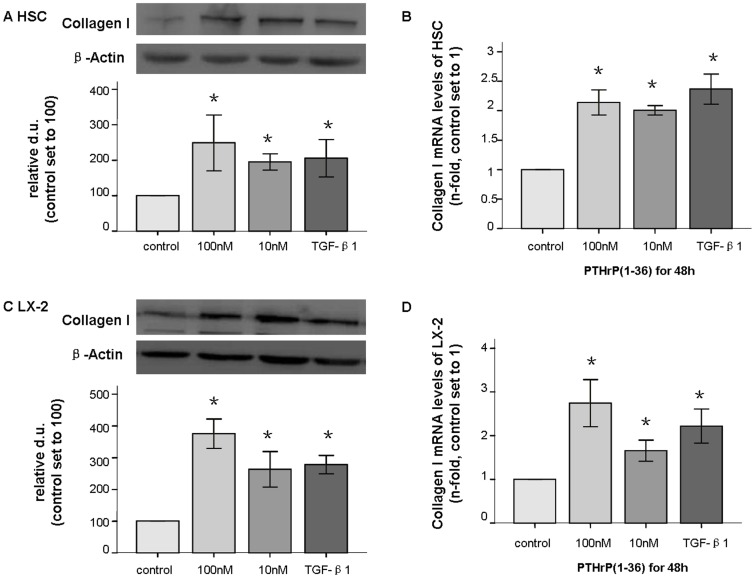
Increased production of collagen I in HSC and LX-2 cells after treatment with PTHrP(1–36). After treatment with PTHrP(1–36) (100 nM) for 48 h, collagen I (138 kd) protein levels were assessed by western-blot (A); mRNA expression was measured with q-PCR and found to be increased in both HSCs (B), and LX-2 cells (C and D). * p<0.05 compared to the control group.

**Figure 4:**
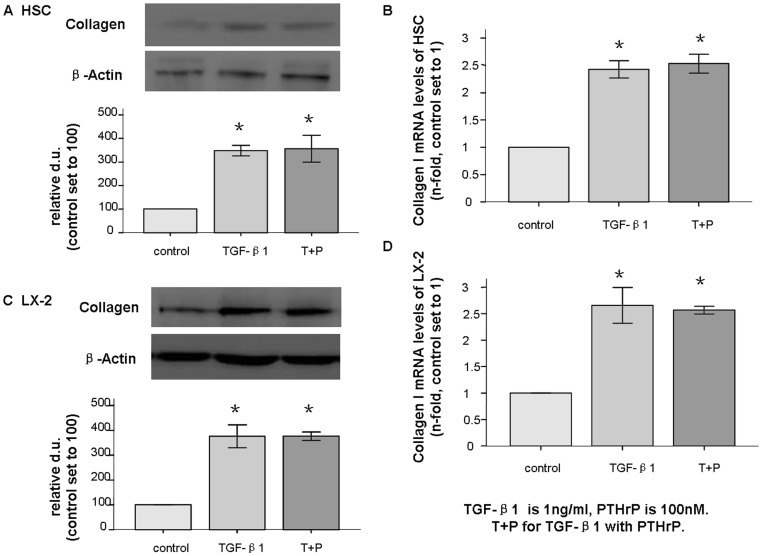
Collagen I expression in HSCs and LX-2 cells after TGF-β1 and PTHrP treatment. After treatment with TGF-β1 (1 ng/ml) alone or with PTHrP (100 nM) for 48 h, collagen I (138 kd) total protein was assessed by western blot (A); mRNA expression was measured with q-PCR and found to be increased in both HSCs (B), and LX-2 cells (C and D). * p<0.05 compared to the control group. No significant difference was observed between treated groups.

Collagen I is the main component of the ECM, and activated HSCs are a major source of collagen type I[12]. In response to PTHrP(1–36) at 10–100 nM for 24–48 h, the collagen I protein (138 kd) was significantly increased 2- to 2.5-fold in HSCs, and its mRNA levels also increased by 2- to 2.5-fold as assessed by real-time PCR compared to untreated control cells (Figure 3). In LX-2 cells, exposure to PTHrP (1–36) at 10–100 nM for 48 h, stimulated collagen I protein and mRNA levels increased by 1.8- to 4-fold, while treatment with PTHrP for 24 h at 100 nM made these increases statistically significant (Figure 3). The other concentrations and time points failed to reach statistical significance in both cell types compared to the control groups. The comparison of TGF-β1 alone to TGF-β1 with PTHrP was not statistically significant with regard to collagen I expression (Figure 4).

**Figure 5:**
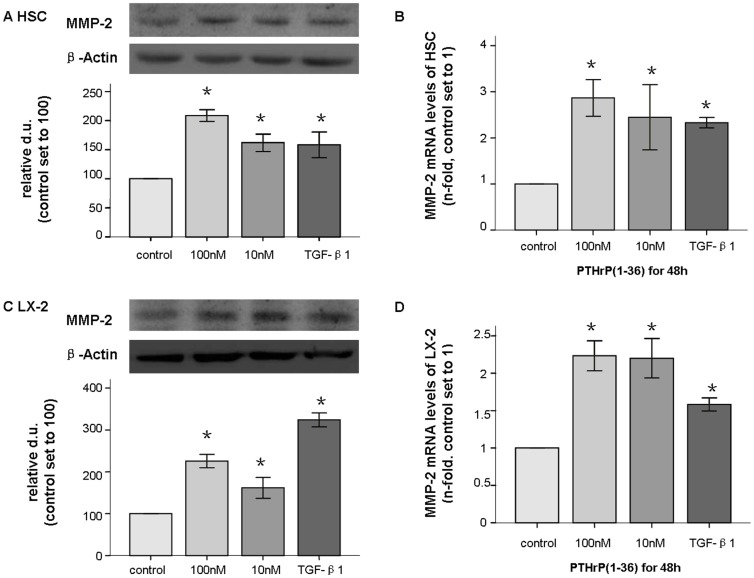
Increased production of MMP-2 in HSCs and LX-2 cells after PTHrP(1–36) treatment. After a 48(1–36), the MMP-2 (72 kd) total protein levels were assessed by western blot (A); mRNA expression was measured with q-PCR and found to be increased in HSC (B). Similar patterns were observed for LX-2 cells (C and D). * p<0.05 compared to the control group.

MMP expression is increased in liver fibrosis, and MMP-2 is expressed and secreted by activated HSCs[20]. After PTHrP was provided at 10–100 nM for 24–48 h, western-blot analysis of whole cell lysates demonstrated that MMP-2 protein (72 kd) levels were increased in both HSC and LX-2 cells by 1.5- to 3-fold. Similarly, we observed increased expression of MMP-2 mRNA by 1.5- to 3-fold in both cell types as assessed by comparative real time PCR (Figure 5). The other concentrations and time points did not show significant differences with regard to MMP-2 levels in either cell type. At least three independent experiments were used for the statistical analyses.

### PTHrP(1-36) induced TGF-β1 secretion in HSC and LX-2 cells

**Figure 6:**
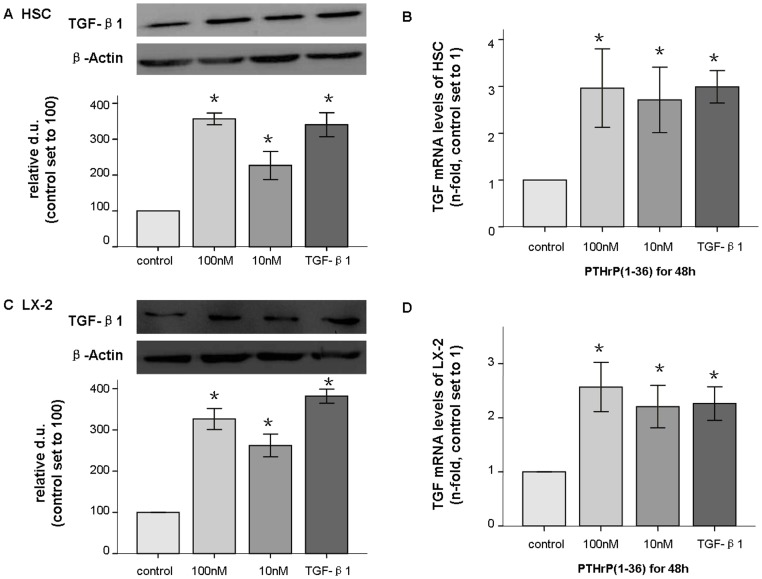
PTHrP(1–36) induced TGF-β1 in HSC and LX-2 cells. After a 48(1–36), TGF -β1 (45 kd) total protein levels were assessed by western-blot (A); mRNA expression was measured with q-PCR and found to be increased in HSC (B), and LX-2 (C and D). * p<0.05 compared to the control group.

**Figure 7:**
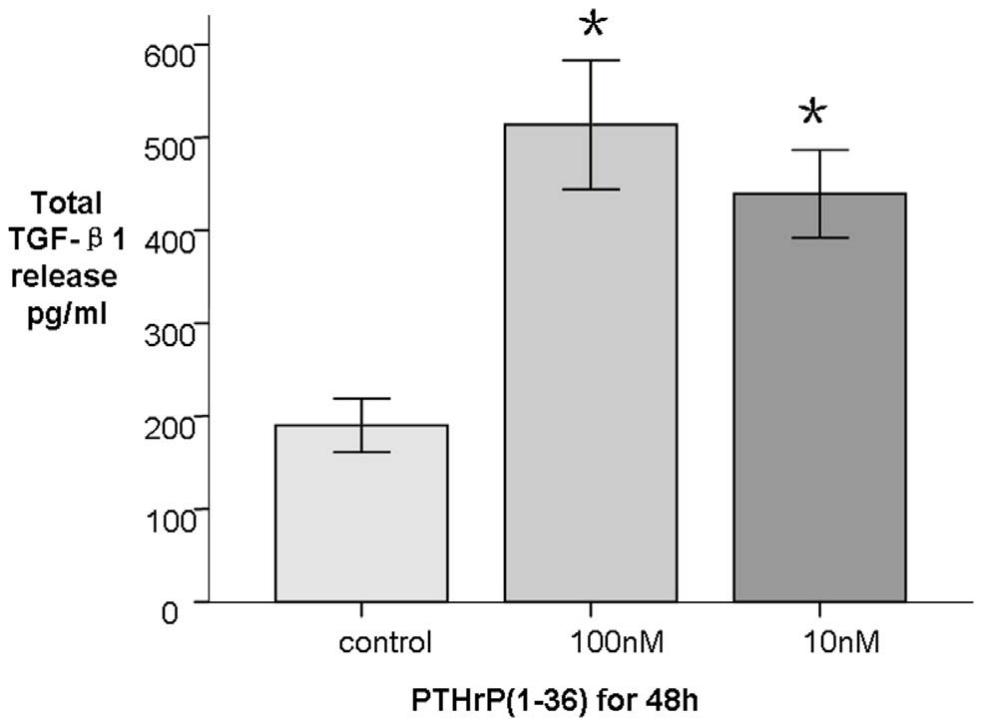
TGF-β1 secretion by HSC after treatment with PTHrP(1–36). Total TGF -β1 secretion in cell culture medium after treatment with 100 nM PTHrP(1–36) for 48 h. * p<0.05 compared to the control group.

It is well accepted that TGF-β1 is a cytokine that plays a central role in fibrosis, and activated HSCs themselves can secrete TGF-β1. We evaluated whether PTHrP would affect TGF-β1 production. We found that TGF-β1 protein (45 kd) in HSCs was stimulated by PTHrP(1–36) at 10–100 nM concentrations as early as 24 h, and 100 nM treatment of LX-2 cells at 48 h, with both cell types exhibiting 2- to 3.5-fold changes. TGF-β1 mRNA levels increased by 2- to 3-fold (Figure 6). TGF-β1 secretion was also measured in HSC cultured medium by ELISA. As expected, this factor was elicited by PTHrP(1–36) at concentrations of 10–100 nM for 24–48 h by 2- to 3-fold (Figure 7). These data suggest that TGF-β1 in HSCs is stimulated by PTHrP(1–36). At least three independent experiments were used for the statistical analyses.

## Discussion

PTHrP was initially identified in cancers that caused lethal paraneoplastic humoral hypercalcemia [1]. PTHrP has a similar structure with parathyroid hormone (PTH), in term of N-terminal amino acid sequence homology and that fact that its full biological activity is contained within the first 34 amino acids [21]. In the previous organ-focused investigations, PTHrP was found to be produced in almost every tissue and organ in the body, including heart, brain, skeletal muscle, bladder, lung, bile duct, immune system, liver, uterus, and testes, as well as most endocrine organs including the pituitary, thyroid gland C-cells, and gastric mucosa enterochromaffin-like cells [14], [15], [22]. As early as 1996, it was reported that PTHrP gene expression was induced in rat vital organs, including liver, spleen, heart, lung, and kidney in response to LPS (lipopolysaccharide) injection [17], [23]. Hepatic PTHrP mRNA levels were acutely induced in rat liver in response to a near lethal dose of endotoxin (LPS), and its protein production was also markedly induced in periportal hepatocytes [16]. It was already established that endotoxins are a critical cofactor in acute and chronic liver disease in both experimental and clinical settings, and their levels correlated with disease severity [19]. These findings suggest that PTHrP may be an additional cytokine involved in liver disease. However, the exact effects of PTHrP in liver cells had not been evaluated.

PTHrP was recently demonstrated to promote fibrogenesis in the obstructed mouse kidney, and it seemed to act in conjunction with TGF-β1, EGF, and VEGF [6], [24]. In our study, we first examined what effects PTHrP might exert in two commercial cell lines. We detected that the activated marker α-SMA was up-regulated by PTHrP treated of HSCs, which was strongly suggestive of activation. Collagen I is a composition of ECM, and MMP-2 degrades the ECM. The previous two factors are mainly produced by activated HSCs and further activate stellate cell growth [7]. In fact, we observed that both collagen I and MMP-2 were increased in PTHrP treated cells compared with control HSCs. Therefore, PTHrP seems to be related to activation changes in HSCs.

LX-2 cells are an activated line that expresses increased levels of α-SMA in contrast to normal stellate cells [8]. However, we still observed increased α-SMA immunoreactivity in PTHrP treated cells by immunofluorescence. We also found increased induction of collagen I, MMP-2, and TGF-β1 mRNA and protein levels in LX-2 cells, but the stimulated effects of collagen I required higher PTHrP concentrations and longer time periods; 10 nM PTHrP treatment for 24 h did not have statistically significant effects. Our results suggest that PTHrP is likely to regulate fibrogenesis by affecting LX-2 cells. However, an in vivo study of human plasma concentrations of PTHrP did not shown an increase in the presence of hepatic cirrhosis, even with severe cases [25]. It is well known that PTHrP can act in endocrine, intracrine, and paracrine, but especially in the latter [1]. Although plasma PTHrP was not significantly increased in hepatic cirrhosis patients, we still observed HSC and LX-2 activation in vitro. These results suggest that PTHrP probably acts in a paracrine fashion. However, elucidation of this cytokine in the intact liver will require further study.

TGF-β1 plays a central role in liver fibrosis, it can induce differentiation of HSCs into collagen producing myofibroblasts. In turn, activated HSCs themselves can secrete TGF-β1, which increases hepatocytes damage [26]. Our in vitro data strongly support the hypothesis that PTHrP contributes to TGF-β1 over-expression in HSCs. It seems that PTHrP may have mediated its effects through TGF-β1 signaling because PTHrP with TGF-β1 did not induce further up-regulation of collagen I compared to TGF-β1 alone. Thus, the present findings also suggest that the activated effects induced by PTHrP in this setting might be mediated, at least in part, by the TGF-β1 system.

The first recognition of PTHrP was related to calcium metabolism, but is was subsequently found to be a potent anabolic agent and was considered as a potential OP treatment in humans [27], [28]. An important complication of chronic liver disease is osteodystrophy, which includes OP, but the general treatment of OP in chronic liver disease is not satisfactory [29]. PTHrP selectively and rapidly stimulates bone formation and has less adverse effects than other options, making it a potential option in treating OP. Moreover, its safety has been partially evaluated but these tests were mostly focused on calcium metabolism [2], [30]. Data regarding the effects on other organs and tissues are limited. Contrary to the fibrogenesis effects of PTHrP in kidney, it has been suggested that PTHrP increases renal plasma flow but does not regulate systemic hemodynamics in healthy humans [31]. It is unclear what effects would be observed in liver, therefore, more studies are needed.

In summary, we show here for the first time the activating effects of PTHrP in HSCs, and its impact on activated LX-2 cells. Our results suggest a role for the TGF-β1 system as a mediator of these effects and support the notion that PTHrP may act as an additional cytokine in liver disease. Further work will assess the relative mechanisms to the actions of PTHrP in this setting.
